# Insights into the Hepatic Arterial Buffer Response in Late-Onset FGR

**DOI:** 10.3390/jcm14238403

**Published:** 2025-11-27

**Authors:** Aziz Kından, Can Ozan Ulusoy, Aykut Kından, Tuğçe Sırma, Aşkın Evren Güler, İsmail Burak Gültekin, Zehra Vural Yılmaz

**Affiliations:** 1 Department of Perinatology, Ankara Etlik City Hospital, Ankara 06170, Türkiye; canozanulusoy@gmail.com (C.O.U.); zehravural@gmail.com (Z.V.Y.); 2 Department of Obstetrics and Gynecology, Nurdağı State Hospital, Gaziantep 27480, Türkiye; aykutkindan33@hotmail.com; 3 Department of Gynecological Oncology Surgery, İskenderun State Hospital, Hatay 31200, Türkiye; drtugcesirma@hotmail.com; 4 Private Clinic of Obstetrics and Gynecology, Ankara 06170, Türkiye; askinevrenguler@yahoo.com; 5 Department of Obstetrics and Gynecology, Ankara Etlik City Hospital, Ankara 06170, Türkiye; burakgultekin@yahoo.com

**Keywords:** hepatic artery Doppler, fetal growth restriction, HABR, fetal hemodynamics, adverse neonatal outcome

## Abstract

**Objective:** To evaluate hepatic artery Doppler parameters in fetuses with fetal growth restriction (FGR) and to investigate their relationship with composite adverse neonatal outcomes (CANO). **Methods:** This prospective cohort study included 108 pregnancies (54 FGR; 54 appropriate-for-gestational-age controls) between 34 and 37 weeks’ gestation. Hepatic artery (HA), umbilical artery (UA), middle cerebral artery (MCA), and uterine artery Doppler indices were recorded. Logistic regression and ROC analyses were used to determine predictors of FGR and CANO. **Results:** HA pulsatility index (PI), systolic/diastolic ratio, and peak systolic velocity (PSV) were significantly higher in FGR fetuses (*p* < 0.05). In multivariate regression, HA-PI remained independently associated with FGR (aOR 1.74, 95% CI 1.07–2.87, *p* = 0.025). For predicting CANO, HA-PSV was the only independent predictor (aOR 1.05, 95% CI 1.00–1.10, *p* = 0.020). ROC analysis demonstrated moderate discriminative ability for HA-PI (AUC 0.681) and HA-PSV (AUC 0.703). **Conclusions:** Increased HA-PSV in FGR reflects activation of the hepatic arterial buffer response as an adaptive mechanism to maintain hepatic perfusion under hypoxic stress, whereas elevated HA-PI may represent evolving microvascular resistance. Hepatic artery Doppler evaluation may serve as a complementary tool for assessing fetal well-being and identifying fetuses at risk for adverse neonatal outcomes, particularly in late-onset FGR.

## 1. Introduction

Fetal growth restriction (FGR) is a significant obstetric problem, occurring in approximately 5–10% of pregnancies and representing a leading cause of perinatal mortality and morbidity [1,2]. It is defined as the failure of a fetus to reach its genetic growth potential and most often results from placental insufficiency [2,3,4]. About one-third of low-birth-weight infants are affected by true FGR, while the remainder are constitutionally small but healthy [5]. FGR is associated with short-term complications such as intrauterine death, asphyxia, and the need for neonatal intensive care, as well as long-term health consequences including cardiovascular disease, type 2 diabetes, and metabolic syndrome, with effects that often persist into adulthood [6,7].

The definition and classification of FGR are of great importance due to their impact on both perinatal and long-term outcomes. The most current definition was developed by the International Delphi Consensus, and according to this guideline, FGR diagnosed after the 32nd week of gestation is classified as late-onset FGR. Diagnostic criteria for late-onset FGR include an estimated fetal weight (EFW) or abdominal circumference (AC) < 3rd percentile, or an EFW/AC < 10th percentile, accompanied by abnormal Doppler findings. These abnormal Doppler findings include a cerebroplacental ratio (CPR) < 5th percentile and/or an umbilical artery pulsatility index (PI) > 95th percentile [2,8]. This approach is considered a significant step in clinical practice because it considers the hemodynamic adaptation of the fetus rather than relying solely on quantitative parameters.

Within the scope of fetal hemodynamic research in FGR, hepatic circulation is a relatively understudied area. However, the liver is a key organ in intrauterine metabolic programming and may play a critical role in shaping the long-term cardiometabolic consequences of FGR. Experimental studies indicate that hepatic tissue is particularly susceptible to oxidative stress, mitochondrial dysfunction, and epigenetic changes, which may predispose affected individuals to insulin resistance, diabetes, and metabolic syndrome later in life [9,10,11]. Furthermore, metabolomic analyses in infants born with intrauterine growth restriction have revealed persistent impairments in energy and amino acid metabolism, further emphasizing the central role of liver dysfunction in the developmental origins of health and disease [12,13,14].

In the fetal and postnatal liver, the hepatic arterial buffer response (HABR) is a key autoregulatory mechanism that compensates for reduced portal (or umbilical) venous inflow by increasing hepatic arterial flow. Prenatal studies suggest that HABR may be activated in FGR to maintain liver perfusion under chronic hypoxic stress, but data on hepatic artery Doppler in late-onset FGR remain limited [15].

Moreover, the relationship between hepatic artery Doppler indices and short-term neonatal outcomes has not been fully elucidated.

Therefore, in this study, we aimed (i) to compare hepatic artery Doppler indices between fetuses with late-onset FGR and appropriate-for-gestational-age controls, and (ii) to investigate the association between hepatic artery Doppler parameters and composite adverse neonatal outcomes (CANO). We hypothesized that fetuses with late-onset FGR would show Doppler changes compatible with activation of the hepatic arterial buffer response and that higher hepatic artery peak systolic velocity would be associated with an increased risk of CANO.

## 2. Materials and Methods

### 2.1. Study Design and Ethical Approval

This prospective cohort study was conducted at the Perinatology Clinic of Ankara Etlik City Hospital. Approval for the study was obtained from the Scientific Research Evaluation and Ethics Committee of Ankara Etlik City Hospital (Decision No: AEŞH-BADEK-2025-0023, Date: 8 January 2025). Written informed consent was obtained from all participants in accordance with the criteria of the Declaration of Helsinki.

### 2.2. Study Population

This prospective cohort study included pregnant women between 34 + 0 and 37 + 0 weeks of gestation. Only fetuses with abdominal circumference (AC) and/or estimated fetal weight (EFW) below the 3rd centile were eligible for the FGR group. Fetuses with absent or reversed end-diastolic flow in the umbilical artery were excluded.

The control group included gestational-age–matched healthy singleton pregnancies with normal biometry (EFW and AC ≥ 10th centile), normal umbilical, middle cerebral artery Doppler, and no maternal or fetal comorbidities.

Exclusion criteria for both groups were:

- Multiple pregnancy
- Maternal age < 18 or >45 years
- BMI > 35 kg/m^2^
- Structural or chromosomal anomalies
- Preeclampsia, gestational diabetes, or other chronic systemic diseases
- Maternal hypertension, pregestational diabetes, renal/hepatic/endocrine disease
- Smoking, alcohol use, chronic medication use, or history of malignancy

All eligible women presenting during the study period were consecutively enrolled.

### 2.3. Sample Size Consideration

The sample size was not determined a priori. Instead, all eligible patients who presented during the study period and met the predefined inclusion and exclusion criteria were consecutively enrolled. Although a power calculation based on the effect size reported by Jader de Jesus Cruz et al. [15] indicated that 46 participants (23 per group) would be sufficient to achieve 95% power at α = 0.05, our study ultimately included a larger cohort of 108 pregnancies (54 FGR and 54 controls). This increased sample size enhances the precision and reliability of the findings.

### 2.4. Study Outcomes

The primary outcome of the study was to assess hepatic artery Doppler alterations in fetuses with late-onset FGR. The secondary outcome was to evaluate the relationship between hepatic artery Doppler parameters and composite adverse neonatal outcomes (CANO).

### 2.5. Clinical and Neonatal Data Collection

Maternal demographics (age, gravidity, parity, weight, BMI, gestational weight gain), obstetric history, comorbidities, medication use, surgical history, and lifestyle factors were recorded at admission.

Postnatal data included gestational age at birth, birthweight and centiles, mode and indication for delivery, Apgar scores, and NICU admission.

CANO was defined as ≥1 of the following: NICU > 48 h, Apgar < 7 (1 or 5 min), neonatal hypoglycemia, RDS, mechanical ventilation, seizures, IVH, HIE, meconium aspiration, or neonatal death.

### 2.6. Doppler Acquisition Protocol

All Doppler examinations were performed by a single obstetric sonographer (A.K., 5 years of experience) using a GE Voluson S10 ultrasound system with a C1-5-RS convex probe. Measurements adhered to the Delphi Consensus and ISUOG Doppler Guidelines.

**Figure 1:**
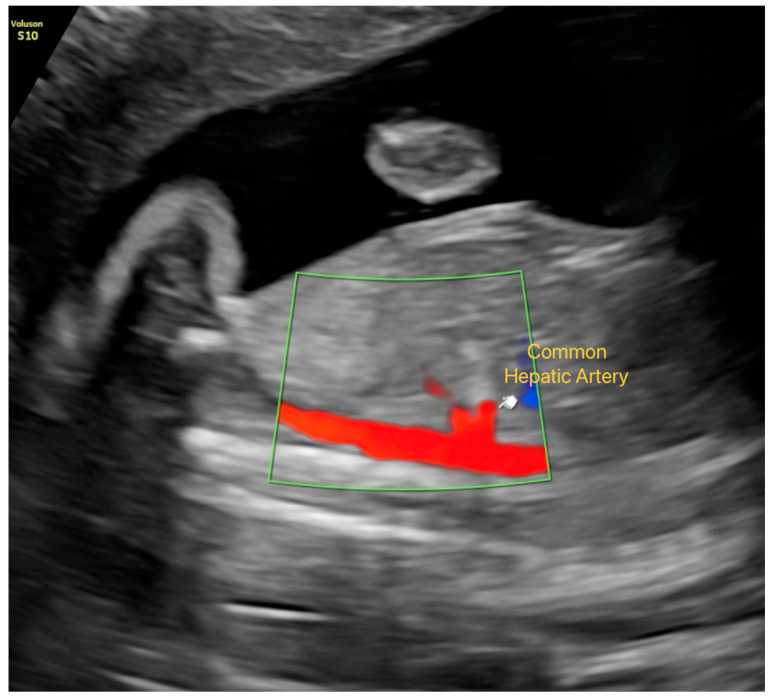
Visualization of the Common Hepatic Artery in Late-Gestation Fetal Ultrasound. Color Doppler imaging showing the common hepatic artery in a sagittal plane immediately distal to its origin from the celiac trunk.

- Umbilical artery (UA): Waveforms were obtained from the free cord segment with an insonation angle < 30°, and PI, RI, and S/D ratio were calculated.
- Middle cerebral artery (MCA): Doppler assessment was performed on the MCA closest to the transducer, sampling the proximal third of the vessel in the axial transthalamic plane.
- Uterine artery: Flow was measured at the level of the cervical canal and internal os, and PI and S/D ratio were recorded.
- Hepatic artery: The common hepatic artery was evaluated in a sagittal plane immediately distal to its origin from the celiac trunk, using the lowest possible angle of insonation. PI, RI, S/D ratio, and peak systolic velocity (PSV) were obtained (Figure 1).

### 2.7. Statistical Analysis

Statistical analyses were performed using IBM SPSS Statistics, Version 29.0 (IBM Corp., Armonk, NY, USA). Continuous variables were analyzed according to normal distribution; parametric data are presented as mean ± standard deviation, and non-parametric data are presented as median (IQR). Student’s *t*-test or Mann–Whitney U test was used for comparisons between groups, and chi-square or Fisher’s exact test was used for categorical variables.

Univariate and multivariate logistic regression analyses were performed for variables associated with FGR and CANO; significant parameters were included in the model using the backward stepwise method. Model fit was assessed using the Hosmer–Lemeshow test, and explanatory power was reported using Nagelkerke R^2^. The predictive power of Doppler parameters was tested using ROC curve analysis; area under the curve (AUC), sensitivity, and specificity values are presented with 95% confidence intervals.

## 3. Results

A total of 108 pregnancies were analyzed, including 54 with fetal growth restriction (FGR) and 54 appropriate-for-gestational-age (AGA) controls. Maternal characteristics were similar between groups (Table 1). At the time of ultrasound, abdominal circumference and estimated fetal weight centiles were significantly lower in FGR (both *p* < 0.001). Postnatally, birthweight and birthweight centiles were markedly reduced (both *p* < 0.001), while NICU admission (40.7% vs. 9.3%, *p* < 0.001) and composite adverse neonatal outcome (CANO) (40.7% vs. 11.1%, *p* < 0.001) were more frequent in the FGR group.

Doppler findings are summarized in Table 2. Umbilical artery PI, S/D ratio, and RI were significantly higher in FGR (all *p* < 0.01), whereas middle cerebral artery PI was reduced (*p* = 0.025) and uterine artery S/D ratio was increased (*p* = 0.028). Notably, hepatic artery PI, S/D ratio, and PSV were also elevated in FGR (all *p* < 0.05).

Logistic regression analyses are shown in Table 3. In univariate analysis, umbilical artery PI (OR 55.30, 95% CI 5.85–522.04, *p* < 0.001) and hepatic artery PI (OR 2.18, 95% CI 1.35–3.50, *p* = 0.001) were significantly associated with FGR. In the multivariate model, umbilical artery PI (aOR 29.61, 95% CI 3.14–279.09, *p* = 0.002), middle cerebral artery PI (aOR 0.55, 95% CI 0.32–0.94, *p* = 0.029), and hepatic artery PI (aOR 1.74, 95% CI 1.07–2.87, *p* = 0.025) remained independent predictors. Model calibration was adequate (Hosmer–Lemeshow *p* = 0.909), and explained variance was moderate (Nagelkerke R^2^ = 0.376).

Associations with CANO are presented in Table 4. Univariate analysis identified umbilical artery PI (*p* = 0.040), middle cerebral artery PI (*p* = 0.033), and hepatic artery PSV (*p* = 0.009) as significant predictors. In the multivariate model, hepatic artery PSV remained independently associated with CANO (aOR 1.05, 95% CI 1.00–1.10, *p* = 0.020). Model calibration was acceptable (Hosmer–Lemeshow *p* = 0.489), although explained variance was modest (Nagelkerke R^2^ = 0.151).

ROC analysis is shown in Table 5. Umbilical artery PI demonstrated the highest predictive performance for FGR (AUC 0.738, sensitivity 61%, specificity 74%). Hepatic artery PI had moderate discriminative ability (AUC 0.681), whereas middle cerebral artery PI (AUC 0.626) and uterine artery PI (AUC 0.422) showed poor predictive value.

## 4. Discussion

The fetal vascular system possesses unique redistribution mechanisms to ensure perfusion of vital organs such as the brain, heart, and adrenal glands from blood carried to the fetus via the umbilical vein. These hemodynamic adaptations become even more important in conditions of chronic stress, such as fetal growth restriction (FGR).

Arterial flow in the adult liver has been shown to be regulated by two primary intrinsic mechanisms. The first is classical arterial autoregulation, which involves myogenic constriction of the hepatic artery when arterial pressure rises. The second, called the hepatic arterial buffer response (HABR), refers to the ability of the hepatic artery to reverse flow regulation in response to changes in portal vein flow. In other words, when portal flow decreases, the hepatic artery dilates, increasing flow; when portal flow increases, it constricts, decreasing flow. The increase in hepatic artery flow following decreased portal vein flow was first observed by Burton-Opitz (1911) [16], and this relationship was defined as “HABR” by Lautt (1981) [17,18,19].

Prenatal studies indicate that HABR is activated in FGR to maintain fetal liver perfusion. When umbilical vein flow decreases, the hepatic artery reduces resistance through compensatory vasodilation, increasing hepatic arterial blood flow [20,21]. Ebbing et al. have shown that hepatic artery PI values decrease at the splanchnic level and liver perfusion redistributes in cases of IUGR. This pathophysiology is supported by findings such as decreased liver oxygenation by umbilical blood, partial substitution by portal flow, and liver hypoperfusion [20]. However, our study showed that HA-PI values increase along with hepatic artery peak systolic velocity (HA-PSV) in fetuses with FGR. Previous studies by Cruz and colleagues provide important evidence supporting the activation of HABR in fetuses exposed to hemodynamic imbalance. In both twin-to-twin transfusion syndrome (TTTS) and selective fetal growth restriction (sFGR), HA-PSV was shown to be significantly increased in the donor or growth-restricted fetus, reflecting compensatory vasodilation and enhanced hepatic arterial inflow. In their TTTS cohort, the HAV-ratio (recipient/donor PSV) was markedly reduced, while the HAPI-ratio also showed a downward trend, although not statistically significant. Although individual HA-PI values were not provided, this pattern suggests a relative increase in donor fetal hepatic artery PI, a finding that aligns with the HA-PI elevation observed in our late-onset FGR cohort. Similarly, in sFGR twins, Cruz et al. demonstrated a significant decrease in the HAV-ratio driven by increased HA-PSV in the growth-restricted fetus, again supporting augmented HABR activity. Taken together, these studies reinforce the concept that FGR, whether in singleton or monochorionic twin pregnancies, is associated with increased hepatic arterial flow and altered resistance indices, consistent with the compensatory mechanisms we observed in our study [22,23].

This discrepancy may be due to the different patient cohort included in our study and the timing of the measurement of hepatic arterial hemodynamic responses, the severity of FGR, and HA hemodynamic values. Hemodynamic deterioration in FGR follows a progressive course over time; we believe that, following a compensation period, distal resistance develops at the microcirculatory level, resulting in a resurgence of PI while PSV remains elevated despite the persistence of HABR. Alexander et al.’s study on rabbit livers showed that under hypoxic conditions (carbon dioxide-free environment), hepatic artery vasodilator responses (acetylcholine, ATP, adenosine, sodium nitroprusside) were significantly reduced. Hepatic artery dilatation, particularly to acetylcholine and ATP, was significantly suppressed. This may be related to microvascular endothelial damage and decreased nitric oxide synthesis [24]. Considering that adenosine is one of the most important mediators of HABR [25], changes in adenosine with advancing weeks of gestation and persistence of chronic hypoxia may also cause changes in the response of the hepatic artery to hypoxia.

In logistic regression analysis, HA-PSV was independently significant in predicting composite adverse neonatal outcome (CANO). This finding suggests that increased HA-PSV may be an indicator of fetal hemodynamic adaptation under hypoxic stress by reflecting increased HABR activity. However, when this compensatory mechanism becomes insufficient, persistently high HA-PSV values may indicate a diminished adaptive response and impending fetal distress. The fact that HA-PSV retains its independent predictive value even after being evaluated in conjunction with other Doppler indices such as umbilical artery PI and middle cerebral artery PI suggests that hepatic artery Doppler may be a complementary indicator in the assessment of fetal well-being. Therefore, the addition of hepatic artery assessment to routine Doppler monitoring, particularly in cases of late-onset FGR, may contribute to the earlier identification of fetuses at risk for adverse perinatal outcomes.

The strengths of our study are its prospective design and the inclusion of a homogeneous population limited to gestational age 34–37 weeks. This approach minimized physiological variability due to gestational age, resulting in more consistent and comparable Doppler measurements. Furthermore, the fact that all Doppler assessments were performed by a single sonographer reduced the risk of interobserver variability and strengthened the internal consistency of the measurements. Including only fetuses with EFW and/or AC below the 3rd percentile, UA Doppler < 95th percentile, and CPR > 5th percentile contributed to a clearer understanding of the hepatic hemodynamic changes characteristic of FGR.

However, the study has several limitations. First, although the study provides a detailed cross-sectional view of hepatic artery hemodynamics in late-onset FGR, the lack of longitudinal follow-up of the same fetus from 32 weeks onward limits our ability to fully assess the dynamic changes in HABR over time. Second, the study was conducted at a single center; although the homogeneous cohort increases internal validity, multicenter studies are needed to confirm the applicability of the findings to larger populations. Another important limitation is that the hepatic artery time-averaged maximum velocity (TAMX) was not measured, which may have limited a more detailed assessment of hepatic arterial hemodynamics.

Future studies should include Doppler measurements of portal system components and right ventricular-atrial pressure gradients to understand the pathophysiological mechanisms. Longitudinal studies combining hepatic artery Doppler with detailed assessment of hemodynamics related to right heart preload and atrial pressure may clarify how redistribution patterns develop and how central venous changes contribute to HABR activation in late-onset FGR. Moreover, beginning in the second trimester, with longitudinal follow-up in FGR may provide a clearer picture of how HABR activity changes over time. Furthermore, the use of advanced imaging modalities such as 3D/4D Doppler or fetal MRI may allow for more detailed assessment of regional differences in liver perfusion and allow for earlier prediction of the risk of liver hypoperfusion associated with FGR.

## 5. Conclusions

This study demonstrates that hepatic artery Doppler, particularly HA-PSV, provides meaningful insight into fetal hemodynamic adaptation in late-onset FGR. Increased HA-PSV supports activation of the hepatic arterial buffer response (HABR), while elevated HA-PI may indicate progressing microvascular resistance. These findings suggest that incorporating hepatic artery assessment into routine Doppler surveillance may enhance risk stratification and perinatal management in late-onset FGR.

## Figures and Tables

**Table 1:** Baseline characteristics, antenatal ultrasound findings, and postnatal outcomes in pregnancies complicated by fetal growth restriction (FGR) compared with appropriate-for-gestational-age (AGA) controls.

	FGR n: 54	AGA n: 54	*p* Value
Demographics, median (IQR)—n (%)			
Maternal age in years	31.0 (24.0, 38.0)	30.0 (24.0, 36.0)	0.584
Parity	2.0 (1.0, 2.7)	2.0 (1.0, 3.0)	0.822
Nulliparity	31 (57.4)	28 (51.9)	0.562
Maternal body mass index at booking in Kg/m^2^	27.0 (25.0, 31.0)	28.0 (26.0, 31.0)	0.672
Antenatal Findings, median (IQR)			
Gestational age at scan (weeks)	35.0 (34.0, 36.0)	34.0 (33.0, 36.0)	0.065
Abdominal Circumference (centile)	1.0 (1.0, 1.0)	36.5 (25.0, 45.8)	**<0.001**
Estimated Fetal Weight (centile)	1.0 (1.0, 1.0)	39.0 (28.3, 48.3)	**<0.001**
Postnatal findings, median (IQR)—n (%)			
Gestational age at birth (weeks)	37.0 (36.0, 37.0)	38.0 (37.0, 39.0)	0.231
Birthweight in grams	2270 (2040, 2458)	3075 (2830, 3408)	**<0.001**
Birthweight (centile)	1.0 (1.0, 1.0)	34.0 (23.3, 47.5)	**<0.001**
APGAR score < 7	4 (7.4)	1 (1.9)	0.169
NICU admission	22 (40.7)	5 (9.3)	**<0.001**
CANO	22 (40.7)	6 (11.1)	**<0.001**

Continuous variables are presented as median (interquartile range, IQR) and categorical variables as number (percentage). Comparisons between groups were performed using the Mann–Whitney U test and Student T test for continuous variables and the χ^2^ or Fisher’s exact test for categorical variables, as appropriate. Composite adverse neonatal outcome (CANO) was defined as the occurrence of one or more of the following: admission to the neonatal intensive care unit > 48 h, Apgar score < 7 at 5 min, neonatal respiratory distress syndrome, need for mechanical ventilation, seizures, intraventricular hemorrhage, hypoxic–ischemic encephalopathy, meconium aspiration syndrome, or neonatal death. A *p*-value < 0.05 was considered statistically significant. FGR: Fetal growth restriction, AGA: Appropriate-for-gestational-age, NICU: Neonatal intensive care unit, IQR: Interquartile range, CANO: Composite Adverse Neonatal Outcome.

**Table 2:** Comparison of Doppler indices between pregnancies with fetal growth restriction (FGR) and appropriate-for-gestational-age (AGA) controls.

	FGR n: 54	AGA n: 54	*p* Value
Umblical Artery, median (IQR)			
Pulsatility Index (PI)	1.02 (0.87, 1.21)	0.85 (0.74, 0.94)	**<0.001**
Systolic/Diastolic Ratio (S/D Ratio)	2.71 (2.38, 3.25)	2.45 (2.21, 2.77)	**0.003**
Resistance Index (RI)	0.64 (0.59, 0.72)	0.59 (0.54, 0.52)	**<0.001**
Middle Cerebral Artery, median (IQR)			
Pulsatility Index (PI)	1.50 (1.31, 2.10)	1.71 (1.51, 2.70)	**0.025**
Systolic/Diastolic Ratio (S/D Ratio)	3.71 (2.96, 4.33)	3.92 (2.43, 4.51)	0.909
Resistance Index (RI)	0.76 (0.72, 1.00)	0.79 (0.75, 1.00)	0.148
Uterin Artery, median (IQR)			
Pulsatility Index (PI)	0.70 (0.53, 1.06)	0.60 (0.44, 0.85)	0.163
Systolic/Diastolic Ratio (S/D Ratio)	1.86 (1.61, 2.80)	1.71 (1.53, 2.10)	**0.028**
Resistance Index (RI)	0.48 (0.38, 0.64)	0.42 (0.36, 0.60)	0.193
Hepatic Artery, median (IQR)			
Pulsatility Index (PI)	2.15 (1.38, 2.88)	1.33 (1.08, 2.18)	**<0.001**
Systolic/Diastolic Ratio (S/D Ratio)	3.16 (2.56, 3.93)	2.75 (2.10, 3.50)	**0.033**
Resistance Index (RI)	0.97 (0.70, 1.28)	1.00 (0.71, 1.31)	0.512
Peak Systolic Velocity (cm/s)	29.3 (26.8, 35.2)	14.6 (13.2, 18.3)	**<0.001**

Values are presented as median (interquartile range, IQR). A *p*-value <0.05 was considered statistically significant. IQR: Interquartile range.

**Table 3:** Logistic regression analysis of Doppler indices for the prediction of fetal growth restriction (FGR).

	Univariate LR		Multivariate LR	
	OR (95% CI)	*p* Value	aOR (95% CI)	*p* Value
Umblical A. PI	55.30 (5.85–522.04)	**<0.001**	29.61 (3.14–279.09)	**0.002**
Middle Cerebral A. PI	0.67 (0.42–1.07)	**0.092**	0.55 (0.32–0.94)	**0.029**
Uterin A. PI	0.83 (0.49–1.40)	0.493		
Hepatic A. PI	2.18 (1.35–3.50)	**0.001**	1.74 (1.07–2.87)	**0.025**

Univariate LR: Univariate logistic regression; Multivariate LR: Multivariate logistic regression; OR: Odds ratio; aOR: Adjusted odds ratio; CI: Confidence interval; PI: Pulsatility index. Univariate associations were evaluated with logistic regression. The multivariable model was constructed using backward stepwise selection (likelihood-ratio) from the four Doppler indices; variables with *p* < 0.05 were retained in the final model. Values are odds ratios (OR) with 95% confidence intervals; bold *p*-values indicate statistical significance (*p* < 0.05). Model calibration was adequate (Hosmer–Lemeshow test *p* = 0.909), and the explained variance was moderate (Nagelkerke R^2^ = 0.376).

**Table 4:** Logistic regression analysis of Doppler indices for the prediction of composite adverse neonatal outcome (CANO).

	Univariate LR		Multivariate LR	
	OR (95% CI)	*p* Value	aOR (95% CI)	*p* Value
Umblical A. PI	4.23 (1.06–16.81)	**0.040**		
Middle Cerebral A. PI	0.48 (0.24–0.94)	**0.033**	0.51 (0.25–1.05)	**0.070**
Uterin A. PI	0.75 (0.37–1.53)	0.439		
Hepatic A. PI	1.35 (0.89–2.05)	0.150		
Hepatic A. PSV	1.06 (1.01–1.11)	**0.009**	1.05 (1.00–1.10)	**0.020**

Univariate LR: Univariate logistic regression; Multivariate LR: Multivariate logistic regression; OR: Odds ratio; aOR: Adjusted odds ratio; CI: Confidence interval; PI: Pulsatility index; PSV: Peak systolic velocity. Univariate associations were evaluated with logistic regression. The multivariable model was constructed using backward stepwise selection (likelihood-ratio) from the four Doppler indices; variables with *p* < 0.05 were retained in the final model. Values are odds ratios (OR) with 95% confidence intervals; bold *p*-values indicate statistical significance (*p* < 0.05). Model calibration was adequate (Hosmer–Lemeshow test *p* = 0.489), and the explained variance was moderate (Nagelkerke R^2^ = 0.151).

**Table 5:** ROC curve analysis of Doppler indices for the prediction of fetal growth restriction (FGR).

	Sensitivity	Specifity	AUC
Hepatic Artery PI			0.681
Cut-off 1.345	51%	76%	
Cut-off 1.410	53%	75%	
Cut-off 1.770	64%	63%	
Cut-off 1.935	70%	58%	
Umblical A. PI	61%	74%	0.738
Middle Cerebral A. PI	70%	42%	0.626
Uterin A. PI	63%	24%	0.422
CPR	73%	55%	0.709
Umblical A. PI	61%	74%	0.738

## Data Availability

Due to hospital policies, patient data and study materials cannot be shared. However, the data are available from the corresponding author upon reasonable request.

## References

[1] Lees C., Marlow N., Arabin B., Bilardo C.M., Brezinka C., Derks J.B.,  Duvekot J., Frusca T., Diemert A., Ferrazzi E. (2013). Perinatal Morbidity and Mortality in Early-Onset Fetal Growth Restriction: Cohort Outcomes of the Trial of Randomized Umbilical and Fetal Flow in Europe (TRUFFLE). Ultrasound Obstet. Gynecol..

[2] Gordijn S.J., Beune I.M., Thilaganathan B.T.A., Papageorghiou A., Baschat A.A., Baker P.N.,  Silver R.M., Wynia K., Ganzevoort W. (2016). Consensus Definition of Fetal Growth Restriction: A Delphi Procedure. Ultrasound Obstet. Gynecol..

[3] Ulusoy C.O., Ağaoğlu R.T., Sucu S.T., Kurt D.S., Bucak M., Şeyhanli Z., Yücel K.Y. (2025). Evaluation of Anterior and Middle Brain Structures With Cerebrovascular Flow in Fetuses With Fetal Growth Restriction: A Prospective Study. J. Clin. Ultrasound.

[4] Akbulut Ö.V., Ağaoğlu R.T., Ulusoy C.O., Kından A., Çanga K., Vural Yılmaz Z. (2025). Prognostic value of foramen ovale morphology and hemodynamics in late-onset fetal growth restriction: A 3D ultrasonography-based study. BMC Pregnancy Childbirth.

[5] Figueras F., Gratacós E. (2014). Update on the Diagnosis and Classification of Fetal Growth Restriction and Proposal of a Stage-Based Management Protocol. Fetal Diagn. Ther..

[6] Crispi F., Miranda J., Gratacós E. (2018). Long-Term Cardiovascular Consequences of Fetal Growth Restriction: Biology, Clinical Implications, and Opportunities for Prevention of Adult Disease. Am. J. Obstet. Gynecol..

[7] Lopian M., Prasad S., Segal E., Dotan A., Ulusoy C.O., Khalil A. (2025). Prediction of Small-for-Gestational Age and Fetal Growth Restriction at Routine Ultrasound Examination at 35–37 Weeks’ Gestation. Ultrasound Obstet. Gynecol..

[8] Lees C.C., Stampalija T., Baschat A., da Silva Costa F., Ferrazzi E., Figueras F., Hecher K., Kingdom J., Poon L.C., Salomon L.J. (2020). ISUOG Practice Guidelines: Diagnosis and Management of Small-for-Gestational-Age Fetus and Fetal Growth Restriction. Ultrasound Obstet. Gynecol..

[9] Cianfarani S., Agostoni C., Bedogni G., Berni Canani R., Brambilla P., Nobili V., Pietrobelli A. (2012). Effect of Intrauterine Growth Retardation on Liver and Long-Term Metabolic Risk. Int. J. Obes..

[10] Oke S.L., Hardy D.B. (2021). The Role of Cellular Stress in Intrauterine Growth Restriction and Postnatal Dysmetabolism. Int. J. Mol. Sci..

[11] Pendleton A.L., Wesolowski S.R., Regnault T.R.H., Lynch R.M., Limesand S.W. (2021). Dimming the Powerhouse: Mitochondrial Dysfunction in the Liver and Skeletal Muscle of Intrauterine Growth Restricted Fetuses. Front. Endocrinol..

[12] Sharma D., Sharma P., Shastri S. (2017). Genetic, Metabolic and Endocrine Aspect of Intrauterine Growth Restriction: An Update. J. Matern. Fetal Neonatal Med..

[13] Gurugubelli Krishna R., Vishnu Bhat B. (2018). Molecular Mechanisms of Intrauterine Growth Restriction. J. Matern. Fetal Neonatal Med..

[14] Esrefoğlu M., Selek S., Koktasoglu F., Bayindir N., Hekimoglu E.-R., Kirmizikan S., Karakaya-Cimen F.-B., Dulun-Agac H., Alim M., Elibol B. (2025). Unraveling Hepatic Consequences of Intrauterine Growth Restriction and Catch-up Growth: Insights from Histological, Biochemical and Metabolomic Analysis in Rats. Int. J. Dev. Biol..

[15] Acar T.T., Asker L., Guven H.K., Kurt G.Y., Ulusoy C.O., Yilmaz E., Yilmaz Z.V. (2025). Fetal Hepatic Artery Doppler in Pregnant Women With Gestational Diabetes Mellitus. J. Clin. Ultrasound.

[16] Burton-Opitz R. (1911). The Vascularity of the Liver. Ii. the Influence of the Portal Blood-Flow Upon the Flow in the Hepatic Artery. Q. J. Exp. Physiol..

[17] Lautt W.W., Legare D.J., Ezzat W.R. (1990). Quantitation of the Hepatic Arterial Buffer Response to Graded Changes in Portal Blood Flow. Gastroenterology.

[18] Jakab F., Ráth Z., Schmal F., Nagy P., Faller J. (1995). The Interaction between Hepatic Arterial and Portal Venous Blood Flows; Simultaneous Measurement by Transit Time Ultrasonic Volume Flowmetry. Hepatogastroenterology.

[19] Eipel C., Abshagen K., Vollmar B. (2010). Regulation of Hepatic Blood Flow: The Hepatic Arterial Buffer Response Revisited. World J. Gastroenterol..

[20] Ebbing C., Rasmussen S., Godfrey K.M., Hanson M.A., Kiserud T. (2009). Redistribution Pattern of Fetal Liver Circulation in Intrauterine Growth Restriction. Acta Obstet. Gynecol. Scand..

[21] Kessler J., Rasmussen S., Godfrey K., Hanson M., Kiserud T. (2009). Fetal Growth Restriction Is Associated With Prioritization of Umbilical Blood Flow to the Left Hepatic Lobe at the Expense of the Right Lobe. Pediatr. Res..

[22] Cruz J.D., Bernardeco J., Rijo C., Cohen A., Serrano F. (2024). Hepatic Arterial Buffer Response in Monochorionic Twins with Selective Fetal Growth Restriction. J. Perinat. Med..

[23] Cruz J.D., Bernardeco J., Cohen A., Serrano F. (2023). Hepatic Arterial Buffer Response in Monochorionic Diamniotic Pregnancies with Twin-to-Twin Transfusion Syndrome. J. Perinat. Med..

[24] Alexander B., Browse D.J., Benjamin I.S. (1999). Hypoxia Attenuates Hepatic Arterial Vasodilatation and Enhances Portal Venous Vasoconstriction to ATP in the Perfused Rabbit Liver. Eur. J. Pharmacol..

[25] Lautt W.W., Greenway C.V. (1987). Conceptual Review of the Hepatic Vascular Bed. Hepatology.

